# Thermal quantitative sensory testing as a screening tool for cardiac autonomic neuropathy in patients with diabetes mellitus

**DOI:** 10.1002/brb3.2506

**Published:** 2022-02-24

**Authors:** Veronika Potockova, Sarka Mala, Lucie Hoskovcova, Vaclav Capek, Tomas Nedelka, Lucie Riedlbauchova, Daniel Baumgartner, Livie Mensova, Radim Mazanec

**Affiliations:** ^1^ Second Faculty of Medicine Department of Neurology, Motol University Hospital Charles University Prague Czech Republic; ^2^ Second Faculty of Medicine Department of Internal Medicine, Motol University Hospital Charles University Prague Czech Republic; ^3^ Faculty of Biomedical Engineering Czech Technical University in Prague Prague Czech Republic; ^4^ Second Faculty of Medicine Department of Cardiology, Motol University Hospital Charles University Prague Czech Republic

**Keywords:** autonomic nervous system, diabetic neuropathy, small fiber neuropathy

## Abstract

**Introduction:**

Electrophysiological diagnosis of cardiac autonomic neuropathy (CAN) is based on the evaluation of cardiovascular autonomic reflex tests (CARTs). CARTs are relatively time consuming and must be performed under standardized conditions. This study aimed to determine whether thermal quantitative sensory testing (TQST) can be used as a screening tool to identify patients with diabetes at a higher risk of CAN.

**Methods:**

Eighty‐five patients with diabetes and 49 healthy controls were included in the study. Neurological examination, CARTs, TQST, biochemical analyses, and neuropathy symptom questionnaires were performed.

**Results:**

CAN was diagnosed in 46 patients with diabetes (54%). CAN‐positive patients with diabetes had significantly higher warm detection thresholds (WDT) and significantly lower cold detection thresholds (CDT) in all tested regions (thenar, tibia, and the dorsum of the foot). CDT on the dorsum < 21.8°C in combination with CDT on the tibia < 23.15°C showed the best diagnostic ability in CAN prediction, with 97.4 % specificity, 60.9% sensitivity, 96.6% positive predictive value, and 67.3% negative predictive value.

**Conclusion:**

TQST can be used as a screening tool for CAN before CART.

## Graphical abstract

This study aimed to determine whether thermal quantitative sensory testing (TQST) can be used as a screening tool to identify patients with diabetes at a higher risk of cardiac autonomic neuropathy. Eighty‐five patients with diabetes were included in the study. Cold detection threshold (CDT) on the dorsum of the foot < 21.8°C in combination with CDT on the tibia < 23.15°C showed the best diagnostic ability in CAN prediction, with 97.4% specificity, 60.9% sensitivity, 96.6% positive predictive value, and 67.3% negative predictive value. TQST can be used as a screening tool for CAN before CART in patients with diabetes mellitus.

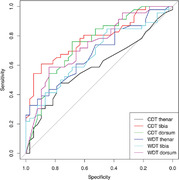

## 1 INTRODUCTION

Cardiac autonomic neuropathy (CAN) is a common and serious complication of diabetes mellitus. In addition to distal symmetric peripheral neuropathy, CAN is one of the most common types of diabetic neuropathy (Pop‐Busui et al., 2017). Patients with diabetes and CAN have higher rates of mortality and morbidity from diseases, such as stroke, coronary artery disease, and silent myocardial ischemia, than patients with diabetes without CAN (Maser & Lenhard, 2005; Spallone et al., 2011; Vinik et al., 2003). CAN diagnosis is based on the evaluation of cardiovascular autonomic reflex tests (CARTs). CARTs are the gold standard for clinical autonomic testing and include assessment of the heart rate response to deep breathing, standing, and the Valsalva maneuver, and the change in blood pressure in response to standing. CARTs are relatively time consuming and must be performed strictly under standardized conditions (Spallone et al., 2011).

Postganglionic autonomic nerves are generally formed from small nonmyelinated C‐type fibers. Postganglionic parasympathetic vagal nerve fibers are typically impaired in CAN. In somatic peripheral nerves, the same C‐type nerve fibers are involved in the perception of warm stimuli and in the slow component of pain. The first studies on thermal quantitative sensory testing (TQST) were published in the 1970s (Dyck et al., 1978; Fruhstorfer et al., 1976). Currently, TQST is an integral part of the diagnosis of somatic small fiber neuropathy in clinical practice within the frame of quantitative sensory testing.

Many recent studies evaluated TQST and CART in patients with diabetes mellitus (Orlov et al., 2012; Tavakoli et al., 2015; Ziegler et al., 2015). In this study, we aimed to determine whether TQST can be used as a screening tool to select patients with diabetes at a higher risk of CAN. The novelty of our study lies in the possibility of using a simple psychophysiological test (TQST) that can help clinicians select a group of patients with diabetes at higher risk of CAN instead of ad hoc use of CARTs, which must be performed strictly under standardized conditions at specialized neurophysiological departments.

## 2 METHODS

### 2.1 Study subjects

A total of 87 patients with diabetes (type 1 diabetes mellitus [T1DM], *n* = 55; type 2 diabetes mellitus [T2DM], *n* = 32) and 52 age‐ and sex‐matched healthy controls were enrolled in this cross‐sectional study. All subjects provided signed informed consent. This study was approved by the local ethics committee. Subjects with other causes of somatic or autonomic neuropathy or serious cognitive impairment (e.g., inability to cooperate during electrophysiological tests) were excluded from the study. Two patients with diabetes (T2DM) and three healthy controls were excluded from the study because of newly detected atrial fibrillation during the CARTs. Ultimately, 85 patients with diabetes (55 T1DM + 30 T2DM patients) and 49 healthy controls completed the study, and their data were analyzed. The main characteristics of the study subjects are summarized in Table 1.

**TABLE 1:** Main characteristics of the study subjects

	Control group	DM	*p* Value
Number of subjects	49	85	
Sex (F/M)	30/19	43/42	.4379
Age (years)	53.9 (12.2)	50.7 (14.5)	.4379
HbA1c (mmol/mol)	38.3 (3.4)	69.9 (19.1)	<.001

DM, diabetes mellitus; M, male; F, female.

*Note*: Values are expressed as mean (standard deviation).

### 2.2 Neurological and clinical examinations and laboratory parameters

All subjects underwent neurological examination that focused on neuropathic sensory and motor symptoms, and the presence of secondary diabetic complications was assessed. The blood pressure after 10 min of rest was measured twice in both the upper extremities using a calibrated tonometer. In addition to clinical status, biochemical analyses, including glucose metabolism, kidney function, liver function, thyroid function, nutritional parameters, and albuminuria, were performed.

The clinical assessment of somatic small nerve fiber function included the sharp/dull pinprick sensation (NeuroTips) and temperature sensation (TipTherm). Large nerve fiber function was assessed based on vibration thresholds using a calibrated 128‐Hz tuning fork, and tactile sensation was assessed with a 10‐g nylon Semmes‐Weinstein monofilament.

### 2.3 TQST and questionnaires

TQST was performed in all subjects. The thermal stimulator (SENSELab‐TERMOTEST MSA, Somedic) was equipped with a 25 × 50 mm thermode (liquid‐cooled Peltier element) with a nonallergic silver contact surface. Cold detection threshold (CDT) and warm detection threshold (WDT) on the thenar eminence, tibia, and lateral aspect of the dorsum of the foot bilaterally were determined. A nonrandomized method of measuring reaction time, that is, the method of limits, was used as an investigative algorithm. The duration of the examination was approximately 20 min. Five values for warm perception and five values for cold perception were recorded in each tested region. The mean of these five values was used as the threshold. Previously published normative data to compare our values of thermal threshold values were used (Magerl et al., 2010).

The following validated questionnaires were completed for all subjects: Michigan neuropathy screening instrument questionnaire (MNSIQ), Michigan neuropathy screening instrument examination (MNSIE), Utah early neuropathy scale (UENS), Survey of autonomic symptoms (SAS), and painDetect (Freynhagen et al., 2006; Herman et al., 2012; Singleton et al., 2008; Zilliox et al., 2011). The presence of neuropathic symptoms was considered if the score of MNSIQ ≥ 4 points, and painful neuropathic symptoms were attributed to patients with painDetect ≥18 points (Freynhagen et al., 2006; Herman et al., 2012). The presence of distal symmetric polyneuropathy (DPN) was examined according to the recommendation of the American Diabetes Association and was determined to subjects with MNSIE score ≥ 2 points (Barbosa et al., 2017; Moghtaderi et al., 2005; Pop‐Busui et al., 2017).

### 2.4 CARTs

The autonomic nervous system was assessed using the Fan study device (Schwarzer, Germany) under standardized conditions (Spallone et al., 2011). The procedures included a deep breathing test (3‐min duration; paced breathing, 6 breaths per min), Valsalva maneuver (against pressure of 40 mmHg for 15 seconds), lying‐to‐standing test (5 min lying, followed by 5 min standing), and blood pressure response during standing to detect orthostatic hypotension. The duration of the tests was approximately 40 min. Time analysis parameters of heart rate variability, including Ewing 30:15 ratio, expiration:inspiration ratio (RR_max_/RR_min)_, and Valsalva ratio, were assessed. Blood pressure response during standing was considered abnormal when the diastolic blood pressure decreased by >10 mmHg or the systolic blood pressure decreased by >30 mmHg within 2 min after standing (Boulton et al., 2005).

According to the American Diabetes Association, CAN can be diagnosed if there is at least one abnormal CART result. The following previously published normative values were used: Ewing ratio > 1.03; expiration:inspiration ratio: age 20−24 years > 1.17, age 25−29 years > 1.15, age 30−34 years > 1.13, age 35−39 years > 1.12, age 40−44 years > 1.10, age 45−49 years > 1.08, age 50−54 years > 1.07, age 55−59 years > 1.06, age 60−64 years > 1.04, age 65−69 years > 1.03, and age 70−75 years > 1.02; and Valsalva ratio > 1.2 (Boulton et al., 2005).

### 2.5 Statistical methods

Statistical analysis was performed using R package version 3.4.4 (R Core Team, 2018, Vienna, Austria). Differences in continuous variables between the studied groups were tested using two‐sample tests. The *t*‐test was used for normally distributed data, and the Mann–Whitney Wilcoxon test for nonnormally distributed data. Differences in categorical variables between the studied groups were tested using the χ^2^ test (twofold contingency table test) or Fisher's exact test. To determine the thermal threshold with the best predictive value for identifying CAN‐positive/negative patients, ROC analysis was performed. The achieved test levels were adjusted for multiple comparisons using the Holm method. Adjusted test levels of <5% were considered statistically significant.

## 3 RESULTS

Patients with diabetes were divided into CAN‐positive and CAN‐negative patients. The main clinical and laboratory parameters of the study groups are presented in Table 2. CAN was diagnosed in 46 patients with diabetes (54%; T1DM, *n* = 32; T2DM, *n* = 14). No statistically significant differences were found in the type and duration of diabetes between the CAN‐positive and CAN‐negative patients (*p* > .05). Compared to normative data, pathological values of CDT were found in 31 CAN‐positive and 8 CAN‐negative patients. Pathological values of WDT were found in 24 CAN‐positive and 6 CAN‐negative patients. The statement “pathological values of CDT and WDT” was determined if at least one abnormal value was measured in at least one tested region.

**TABLE 2:** Clinical and neurophysiological parameters of the control group and patients with diabetes according to CAN presence

				*p* Values		

Parameter	Control group	CAN‐positive patients with diabetes	CAN‐negative patients with diabetes	CAN+ vs. CAN–	CG vs. CAN+	CG vs. CAN–
**Number of subjects**	49	46 (54%)	39 (46%)	N/A		
**Type of diabetes**	N/A	T1DM (*n* = 32)	T1DM (*n* = 23)	1.0000	N/A	N/A
		T2DM (*n* = 14)	T2DM (*n* = 16)			
**Sex (F/M)**	30/19	21/25	22/17	1.0000	.4515	1.0000
**Age (years)**	54 (12.1)	50 (13.1)	51 (15.8)	1.0000	.4515	1.0000
**Duration of DM (years)**	N/A	25.4 (13.4)	17.5 (10.1)	.0702	N/A	N/A
**HbA1c (mmol/mol)**	38.3 (3.4)	71.0 (18.4)	68.5 (20.1)	1.0000	<.001	<.001
**Systolic blood pressure (mmHg)**	126.7 (14.2)	137.3 (17.7)	129.6 (13.9)	.4932	.0081	1.0000
**Diastolic blood pressure (mmHg)**	76.0 (7.3)	81.8 (8.4)	73.5 (8.3)	<.001	.0081	1.0000
**UENS (points)**	0.6 (1.4)	12.5 (9.2)	5.9 (5.8)	.0228	<.001	<.001
**MNSIQ (points)**	0.9 (1.3)	4.8 (2.7)	2.4 (2.2)	.0018	<.001	.0128
**Neuropathic symptoms (pts)**	1 (2%) ≥ 4	31 (67.4%) ≥ 4	14 (35.9%) ≥ 4	.0876	<.001	.0017
**MNSIE (points)**	0.2 (0.4)	4.0 (2.6)	1.8 (2.0)	.0025	<.001	<.001
**PainDetect (points)**	2.8 (3.1)	7.7 (6.9)	4.4 (6.6)	.1030	.0050	1.0000
**Neuropathic pain (pts)**	0	6 (13%) ≥ 18	3 (7.7%) ≥ 18	.6561	.0972	.4537
**SAS—NOS (points)**	2.2 (1.9)	3.5 (1.8)	2.6 (1.8)	.4932	.0081	1.0000
**SAS—TIS (points)**	5.0 (4.8)	8.8 (5.0)	6.6 (5.2)	.4932	.0033	1.0000
**DPN (pts)**	0	37 (80.4%)	20 (51.3%)	.0968	.0081	.0175
**Pathological values of WDT (pts)**		24 (52.2%)	6 (15.4%)	.0224		
**Pathological values of CDT (pts)**		31 (67.4%)	8 (20.5%)	.0020		
**Ewing ratio**	1.32 (0.2)	1.08 (0.09)	1.28 (0.26)	<.001	<.001	.5643
**E:I ratio**	1.23 (0.15)	1.06 (0.05)	1.17 (0.13)	<.001	<.001	.3588
**Valsalva ratio**	1.65 (0.43)	1.24 (0.19)	1.61 (0.39)	<.001	<.001	1.0000
**Postural change in sBP (mmHg)**	–8.7 (10.2)	–8.7 (19.4)	–3.3 (14.1)	1.0000	.5974	.3588

CG, control group; CAN+, CAN‐positive patients with diabetes; CAN–, CAN‐negative patients with diabetes; T1DM, type 1 diabetes mellitus; T2DM, type 2 diabetes mellitus; M, male; F, female; SAS, survey of autonomic symptoms questionnaire; NOS, number of symptoms; TIS, total symptom impact score; DPN, distal symmetric polyneuropathy; WDT, warm detection threshold; CDT, cold detection threshold; E:I, expiration:inspiration; sBP, systolic blood pressure; *n*, number of patients; pts, patients; N/A, not applicable.

*Note*: Values are expressed as mean (standard deviation).

The difference between the number of diabetic patients with pathological thermal thresholds between CAN‐positive and CAN‐negative was statistically significant in CDT (*p* = .0020) and WDT (*p* = .0224).

Further, all measured values of CDT and WDT were compared in CAN‐positive and CAN‐negative patients with diabetes in more detail. CDT values were significantly lower in CAN‐positive patients on the thenar eminence (*p* = .0398), tibia (*p* < .001), and lateral aspect of the dorsum of the foot (*p* < .001). WDT values were significantly higher in CAN‐positive patients on the thenar prominence (*p* = .0205), tibia (*p* = .0205), and lateral aspect of the dorsum of the foot (*p* < .001).

**FIGURE 1:**
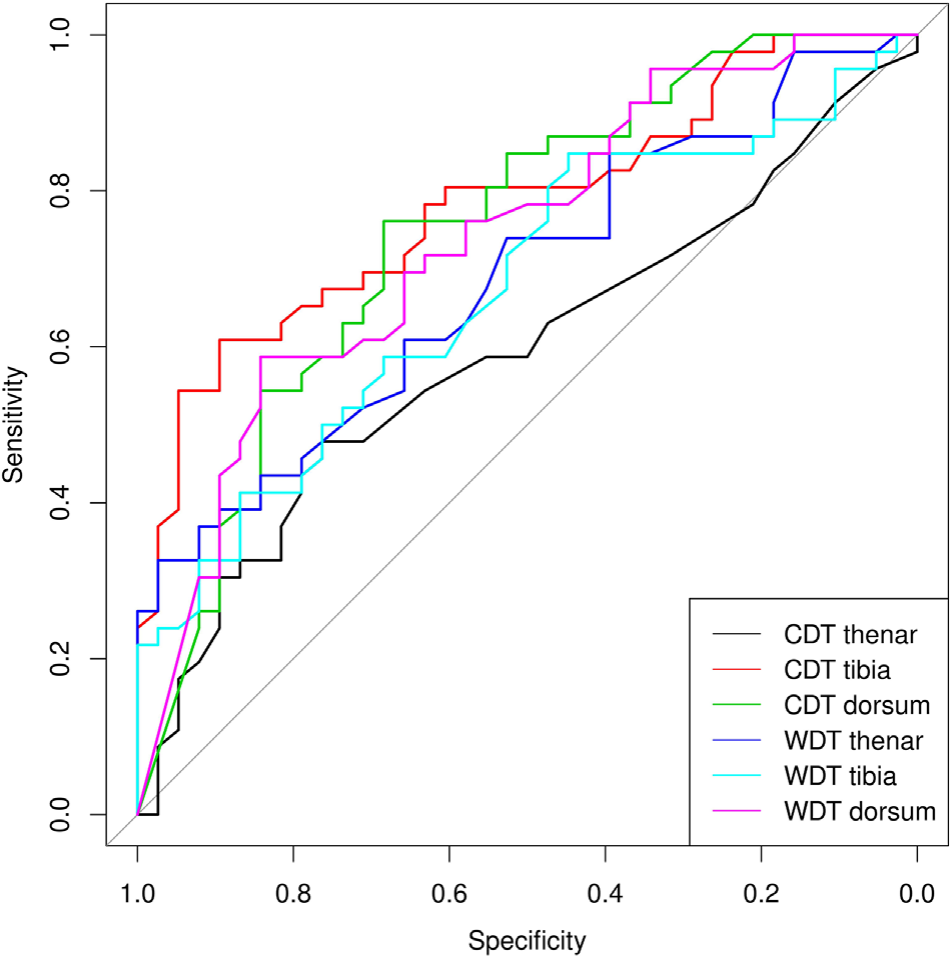
ROC curves for CDT and WDT in predicting CAN. ROC, receiver operating characteristic; CDT, cold detection threshold; WDT, warm detection threshold

ROC analysis for the thermal thresholds on the thenar, tibia, and dorsum of the foot was performed to determine the diagnostic ability in predicting CAN. CDT measured on the dorsum of the foot with the cut‐off value of 21.8°C (AUC .7486, sensitivity 76.1%, specificity 68.4%), CDT measured on the tibia with the cut‐off value of 23.15°C (AUC .7829, sensitivity 60.9%, specificity 89.5%), and WDT measured on the dorsum of the foot with the cut‐off value of 46.35°C (AUC .7374, sensitivity 58.7%, specificity 84.2 %) were tests with the best predictive value. The combination of more TQST parameters in more locations increased the specificity and positive predictive value. CDT on the dorsum of the foot <21.8°C in combination with CDT on the tibia <23.15°C showed the best diagnostic accuracy in the CAN prediction with 97.4% specificity, 60.9% sensitivity, 96.6% positive predictive value, and 67.3% negative predictive value. The results of TQST in predicting CAN in patients with diabetes are summarized in Table 3. ROC curves for CDT and WDT are shown in Figure 1.

**TABLE 3:** ROC analysis for TQST in predicting CAN in patients with diabetes mellitus

TQST	AUC	Cut‐off value	Sensitivity	Specificity	PPV	NPV
**CDT thenar**	.5924	29.35	47.8%	76.3%	71.0%	54.7%
**CDT tibia**	.7829	23.15	60.9%	89.5%	87.5%	65.4%
**CDT dorsum**	.7486	21.80	76.1%	68.4%	74.5%	70.3%
**WDT thenar**	.6868	37.65	32.6%	97.4%	93.8%	54.4%
**WDT tibia**	.6751	40.85	84.8%	44.7%	65.0%	70.8%
**WDT dorsum**	.7374	46.43	58.7%	84.2%	81.8%	62.8%
**Combination** **CDT dorsum** **CDT tibia**		<21.8 <23.15	60.9%	97.4%	96.6%	67.3%
**Combination** **CDT dorsum** **WDT dorsum**		<21.8 >46.35	58.7%	86.8%	84.4%	63.5%
**Combination** **CDT dorsum** **CDT tibia** **WDT tibia**		<21.8 <23.15 >40.85	58.7%	97.4%	96.4%	66.1%

CDT, cold detection threshold; WDT, warm detection threshold; AUC, area under the curve; PPV, positive predictive value; NPV, negative predictive value.

*Note*: Cut‐off values of CDT and WDT are expressed in °C.

In addition to TQST, several biochemical metabolic parameters and questionnaires focused on neuropathic symptoms were also analyzed to identify other potential markers that could contribute to the pathophysiology of CAN in diabetes mellitus.

Statistically significant differences were found between CAN‐positive and CAN‐negative patients (*p* < .05) in the following variables: diastolic blood pressure (*p* < .001), albumin/creatinine ratio (*p* = .002), UENS score (*p* = .0228), MNSIQ score (*p* = .0018), and MNSIE score (*p* = .0025). All of the above variables had higher values in the CAN‐positive patients. The diagnostic accuracy for these variables in predicting CAN was not superior compared to TQST. The highest 80.4% sensitivity but low specificity of 61.5% showed the diastolic blood pressure with the cut‐off point of 76.5 mmHg. MNSIQ with the cut‐off value of 5.5 points was the questionnaire with the highest specificity of 94.9% but poor 50% sensitivity. Complete data are presented in Table 4. No significant difference was found for SAS score (*p* = .4932) and presence of distal symmetric polyneuropathy (*p *= .0968).

**TABLE 4:** ROC analysis for questionnaires, diastolic blood pressure, and albumin/creatinine ratio in predicting CAN in patients with diabetes

Parameter	AUC	Cut‐off value	Sensitivity	Specificity	PPV	NPV
**MNSIQ**	.7581	5.5 points	50.0%	94.9%	92.0%	61.7%
**MNSIE**	.7520	2.75 points	71.7%	69.2%	73.3%	67.5%
**UENS**	.7185	4.5 points	76.1%	59.0%	68.6%	67.7%
**Diastolic BP**	.7522	76.5 mmHg	80.4%	61.5%	71.2%	72.7%
**Alb/crea ratio**	.7477	1.009 g/mol	67.4%	73.7%	75.6%	65.1%

MNSIQ, Michigan neuropathy screening instrument questionnaire; MNSIE, Michigan neuropathy screening instrument examination; UENS, Utah early neuropathy scale; BP, blood pressure; AUC, area under the curve; PPV, positive predictive value; NPV, negative predictive value.

No statistically significant difference (*p* > .05) between CAN‐positive and CAN‐negative patients was found in the following biochemical parameters: fasting blood glucose, glycosylated hemoglobin, triglycerides, cholesterol, low‐ and high‐density lipoprotein, plasma urea and creatinine, thyroid‐stimulating hormone, and free thyroxine.

All study groups did not differ significantly in the postural change of systolic blood pressure during the lying‐to‐standing test and in presence of neuropathic pain.

## 4 DISCUSSION

Our study showed that CAN‐positive patients with diabetes had significantly higher WDT and significantly lower CDT in all tested regions (thenar, tibia, and the dorsum of the foot). Quantitative sensory testing of CDT in the lower extremities showed the best diagnostic ability to differentiate CAN‐positive from CAN‐negative patients. CDT on the dorsum of the foot <21.8°C in combination with CDT on the tibia <23.15°C can predict CAN with a very high specificity of 97.4% and a positive predictive value of 96.6%. This TQST combination showed a lower sensitivity of 60.9% and a negative predictive value of 67.3%, which means some patients with CDTs under these cut‐off values were evaluated as a false negative for the presence of CAN. Detection of a WDT value on the tibia >40.85°C with a high sensitivity of 85% and a moderate negative predictive value of 69% for CAN prediction can help.

Further, CAN‐positive patients scored significantly higher on questionnaires describing somatic neuropathic symptoms such as MNSIQ, MNSIE, and UENS with a sensitivity up to 76% and specificity up to 94.9%. The fact is not surprising since both autonomic and sensory neuropathies are frequently combined (Serhiyenko et al., 2018). Similar high scores on MNSI questionnaire responses in CAN‐positive patients were reported by Islam et al. (2018).

We did not find a correlation between autonomic symptoms questionnaire (SAS) values and the presence of CAN. Similar results were published by Low et al. (2004). Zilliox et al. (2011) showed an association of increased SAS score with only one CART parameter reduced Ewing 30:15 ratio.

In addition to TQST and questionnaires, significantly higher diastolic blood pressure values (cut‐off point 76.5 mmHg) and higher albumin/creatinine ratios (cut‐off point 1.009 g/mol) were measured in patients with CAN. Our results are congruent with previously published studies that demonstrated higher diastolic blood pressure values (Rolim et al., 2008; Azmi et al., 2019; Spallone, 2019) and higher prevalence of microalbuminuria (Astrup et al., 2006; Spallone et al., 2011) in patients with diabetes who suffer from CAN.

Other well‐known risk factors for developing CAN include diabetes duration and poor glycemic control (Serhiyenko et al., 2018). We did not find a significant difference in glycemic control between the patients with CAN and without CAN. Similar results were published by Vasheghani et al. (2019). The duration of diabetes was, on average, longer in our CAN‐positive patients, but the difference was not statistically significant. The above results are probably due to the lower number of study subjects and the evaluation of only a single value of glycosylated hemoglobin for each patient.

Several researchers also tried to predict CAN using simpler tests than CART. Pafili et al. (2020) showed that normal pinprick and thermal sensation in lower extremities assessed using qualitative bedside tests, such as NeuroTips and Tiptherm, yielded a very high negative predictive value (97%) for the diagnosis of CAN in T2DM patients. Yajnik et al. (2013) suggested that the SUDOSCAN could be used as a simple noninvasive screening test for diabetic CAN with a sensitivity of 92% and specificity of 49%. Similar results were published by Selvarajah et al. Their study showed that SUDOSCAN has 65% sensitivity and 80% specificity to diagnose CAN (Selvarajah et al., 2015).

## 5 CONCLUSION

TQST seems to be a potential noninvasive, time‐saving, and relatively simple tool to detect patients with diabetes with a higher risk of CAN. Our study showed that CAN‐positive patients had significantly higher WDT and significantly lower CDT in all tested regions (thenar, tibia, and the dorsum of the foot). The best diagnostic ability in the CAN prediction we found to be CDT on the dorsum of the foot and CDT on the tibia. The study confirmed that TQST can be used as screening tool to identify diabetic patients for further autonomic testing in clinical practice.

## CONFLICT OF INTEREST

The authors do not have any conflicts of interest to declare.

### PEER REVIEW

The peer review history for this article is available at https://publons.com/publon/10.1002/brb3.2506.

## Data Availability

The data that support the findings of this study are openly available in a public repository that issues datasets with DOIs.
